# Novel study design to assess the utility of the copd assessment test in a primary care setting

**DOI:** 10.1186/1471-2288-13-63

**Published:** 2013-05-10

**Authors:** Kevin Gruffydd-Jones, Helen Marsden, Steve Holmes, Peter Kardos, Roger Escamilla, Roberto Dal Negro, June Roberts, Gilbert Nadeau, David Leather, Paul Jones

**Affiliations:** 1 Box Surgery, Box, Corsham, United Kingdom; 2 Respiratory Centre of Excellence, GlaxoSmithKline, London, United Kingdom; 3 The Park Medical Practice, Shepton Mallet, United Kingdom; 4 Gemeinschaftspraxis und Zentrum für Pneumologie, Allergologie, Schlafmedizin, Frankfurt, Germany; 5 Hôpital Larrey- CHU Toulouse, Toulouse, France; 6 Ospedale Civile Orlandi di Bussolengo, Bussolengo, Italy; 7 Salford Royal Hospital Foundation Trust, Salford, United Kingdom; 8 St George's University of London, London, United Kingdom

**Keywords:** COPD, CAT, Primary care consultation, Videoed assessment

## Abstract

The quality of a consultation provided by a physician can have a profound impact on the quality of care and patient engagement in treatment decisions. When the COPD Assessment Test (CAT) was developed, one of its aims was to aid the communication between physician and patient about the impact of COPD. We developed a novel study design to assess this in a primary care consultation.

Primary care physicians across five countries in Europe conducted videoed consultations with six standardised COPD patients (played by trained actors) which had patient-specific issues that the physician needed to identify through questioning. Half the physicians saw the patients with the completed CAT, and half without. Independent assessors scored the physicians on their ability to identify and address the patient-specific issues, review standard COPD aspects, their understanding of the case and their overall performance. This novel study design presented many challenges which needed to be addressed to achieve an acceptable level of robustness to assess the utility of the CAT. This paper discusses these challenges and the measures adopted to eliminate or minimise their impact on the study results.

## Introduction

The quality of a consultation provided by a physician can have a profound impact on the quality of care and patient engagement in treatment decisions [1]. The most effective consultations are those in which doctors most directly acknowledge and respond to patients’ problems and concerns [2]. Limited time for consultations forces primary care physicians to focus on the fundamental problems, and patients often do not present all of their problems and concerns in a consultation, which can lead to poor consultation outcome [3]. Thus, tools to improve the communication between patient and physician have the potential to improve consultation outcomes.

The COPD Assessment Test (CAT) is a new, patient completed questionnaire designed to provide a simple and reliable measure of health status in a patient with COPD [4]. The CAT questionnaire is formed of 8 questions covering the most burdensome symptoms of COPD. The CAT has undergone robust validation testing, and has been shown to have very similar properties to the more complex health status questionnaires, the St George’s Respiratory Questionnaire (SGRQ) [5] and the Chronic Respiratory Questionnaire [6]. However, it is shorter, making it suitable for routine clinical use.

In general, once a patient reported outcome measure (PROM) has been developed and validated, there needs to be an assessment of whether it improves patient care [7]. When the CAT was developed, one of its aims was to aid the communication between physician and patient on the impact of COPD, and thus aid physicians to optimise the patients’ care [4]. However, to date, this aspect of the CAT has not been tested. We therefore set out to conduct a study to assess the impact of a PROM, the CAT, on physician-patient communication.

Such a research question presents several challenges to researchers: How to ensure a fair comparison between arms; how to assess the impact of the PROM; and how to conduct a study large enough (both sample size and geographical spread) to give robust and generalisable results? This paper discusses the feasibility of a novel study which was designed to address many of these challenges.

### Study design

**Figure 1:**
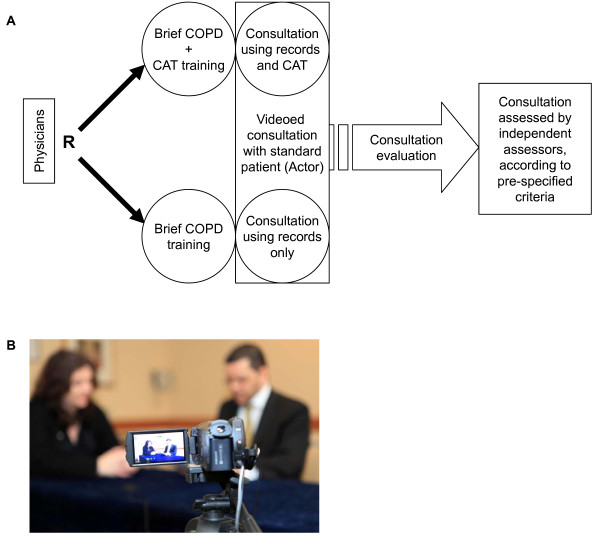
**Study design.** (**A**) Physicians were randomised to conduct consultations with standardised COPD patients either with or without the CAT; (**B**) The physician: patient consultations were videoed for assessment. R: Randomisation; COPD: Chronic Obstructive Pulmonary Disease; CAT: COPD Assessment Test.

This was a single visit, randomised (1:1) open, parallel group study (Figure 1). The UK’s National Research Ethics Services confirmed ethical approval was not required, and physicians consented to their participation in the study.

### Physician recruitment

The CAT had been launched by the time the study commenced and was already being used by some secondary care respiratory specialists. The study therefore focused on the primary care setting to aid recruitment and to assess the CAT where the impact of its use is unknown. Physicians across five European countries (United Kingdom (UK), Ireland, France, Germany and Austria) were contacted by a local market research agency and screened by telephone interview. Those reporting experience of managing COPD patients (at least three), but not of using the CAT, were invited to participate in a physician-patient communication study. A total of 168 physicians agreed to participate in the study, of which three failed to attend their allotted filming session. The selection of the physicians was not random due to the practicalities of the study, which required twelve physicians who were willing to participate in the study and within travelling distance of suitable filming facilities. Potential biases of location (rural versus urban-based physician) and specific interest or expertise in respiratory diseases were considered. Location (rural versus urban within an individual country) is unlikely to affect physician communication skills, but knowledge of COPD and the CAT may have done. The screening questions therefore also covered experience of managing asthma, use of Hospital Anxiety and Depression Score, and Framingham risk such that physicians were unaware that the study was about COPD and the CAT until they attended their allocated sessions. Physicians were asked again about whether they had a working knowledge of the CAT when the attended the filming session to identify any physician who had used the CAT following the screening interview. A total of nine physicians reported experience of using the CAT on the day of filming. These physicians were excluded from the primary analysis, along with any physicians who reported insufficient experience of managing COPD patients on the day of filming.

Two or four geographically-spread locations were used in each country, depending on the number of physicians recruited (Table 1). Physicians attended the sessions in groups of five or six, with two groups recruited in each location. Each group was randomised to see the patient with (“*CAT+*” arm) or without (“*no CAT*” arm) the completed CAT during the consultation. A 2-level hierarchical design was used, with a randomisation block size of two, without stratification, such that one group was randomised to each arm at each location. The recruiters were blinded to the randomisation.

**Table 1:** Breakdown of physicians and assessors by country

	**Physicians**	**Group size**	**Locations**	**Assessors**
Austria	24	6	2	N/A
France	39	5	4	5
Germany	38	5	4	9
Ireland	24	6	2	N/A
UK	40	5	4	10
Total	165	N/A	16	24

N/A: Not applicable.

Physicians received brief training on COPD and those in the *CAT+* arm also received brief training on the CAT. The training was provided in the form of reading material, which for the CAT+ groups included background information on the tool, how to interpret overall scores and how to identify specific areas of concern for the patient. Participants were given around 20 minutes to complete the training, and were encouraged to discuss the information between them. This level of training may be inadequate to provide sufficient understanding for physicians to change their behaviour based on the CAT results, but is often reflective of time and training available to primary care physicians in real life. Practical educational approaches generally encourage more behavioural changes [8], but this would not have been practical to implement in this study. No specific guidelines were provided on actions to take based on the CAT score as such advice was not available at the time.

### Consultations

Physicians then undertook videoed consultations with six standardised COPD patients. Each physician conducted all their consultations either with or without the CAT so that non-CAT consultations were not influenced by questions / practices adopted in consultations with the CAT. After the consultation, physicians were asked to record to camera their impression of the case and recommended course of action – as if they were making notes in the patient’s records.

Because of the practicalities of the study, the physicians were meeting the patient actor for the first and only time, which may have driven a different kind of consultation compared with consecutive consultations. A physician first needs to build a relationship with the patient, and then become acquainted with all his/her diagnoses, of which COPD may be just one (and not always the most important). Additionally, a maximum time was allowed for each consultation, with discussions being interrupted and asked to finish after ten minutes, limiting the time the physicians had to build that relationship. However, this is representative of real-life time pressures in the primary care setting.

Interestingly, during validation work on the Clinical COPD Questionnaire it was noted that physicians changed their practices with experience of using the PROM [9]. If this phenomenon is representative, the utility of CAT may be different when the relationship between the patient and physician is more established, and in which the physician has a better understanding of the patient’s history and situation.

### Standardised patient cases

Pendleton’s tasks [10] are often used to assess the quality of a consultation and have previously been used to assess the communication of a healthcare provider with their patient [11]. The tasks include “understanding the patient issues”, and “involving the patient in the choice of action”. Similar descriptions of what a good consultation should achieve have been described by Howie [12] and Mauksch [13], which include skills such as “topic tracking”, “emphatic response to cues” [13], “patient priorities” and “sharing decision making” [12]. The expected impact of CAT is on the specific COPD content of the discussion, rather than physicians’ values and personality. We therefore decided to assess the ability of the physicians to identify ‘patient issues’, COPD specific issues and management of these issues with and without the CAT. These issues represented aspects of the patients’ medical history that needed to be addressed by the physicians, such as depression, review of lifestyle or therapy, and compliance issues.

**Figure 2:**
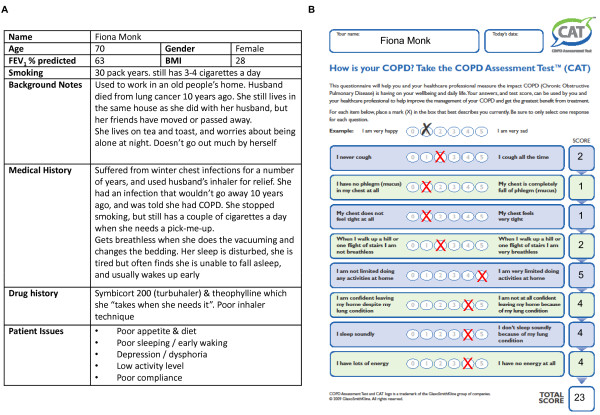
**Example COPD patient case.** (**A**) Each case history was designed to cover a variety of disease severities and scenarios relevant to clinical practice. (**B**) The actors provided completed CAT forms to physicians in the *CAT+* arm. CAT scores for each case were independently verified. FEV_1_: Forced Expiratory Volume in 1 second; BMI: Body Mass Index; COPD: Chronic Obstructive Pulmonary Disease; CAT: COPD Assessment Test.

The authors (including active clinicians from a variety of different environments) constructed six cases to test the utility of CAT across a range of scenarios, disease burden and COPD patient issues (Table 2, Figure 2). Some of the social aspects of each case were amended slightly for each country, to ensure they resonated with the physicians. The CAT scores of each case were independently verified. Each case included four or five ‘patient issues’. While this allowed us to test the utility of the CAT in the areas we believed it may impact, they may not have represented valid patient scenarios, for example the presence of several issues in each case. Alternative methods of creating the standardised cases, such as selecting actual COPD patient cases, and have the actors portray those individual cases would have been equally valid.

**Table 2:** Patient case summaries

**Case**	**Age**	**FEV**_**1**_	**CAT**	**Medical history**	**Patient issues**
**1**	68	40	34	Severe COPD, highly burdened by disease	Mildly depressed; restricted in activities; need for pulmonary rehabilitation
**2**	60	30	21	Sedentary lifestyle, post- severe exacerbation	Loss of confidence; need for lifestyle & therapy review
**3**	50	70	9	Recently diagnosed mild COPD, mild burden of disease	Anxiety of diagnosis; need for lifestyle advice & general COPD management
**4**	65	45	16	CV co-morbidity which being well treated, but poorly managed COPD	Continued smoking & limited exercise; impact of disease on activities; poor compliance
**5**	70	68	23	Severely limited by disease - overt depression	Manifestations of depression; poor compliance; need for pulmonary rehabilitation & social support
**6**	63	65	19	Immigrated from Middle East / North Africa. Suffering bad chest infection, wants antibiotics. Highly burdened by cough	Doesn't believe he has COPD; need to appreciate impact of disease burden

Description of each of the six patient cases. Medical history briefly explains the case that was presented to the physician, while the patient issues are those elements of the case that the physician needed to identify and address. FEV_1_: Forced Expiratory Volume in 1 second; COPD: Chronic Obstructive Pulmonary Disease; CAT: COPD Assessment Test; CV: cardiovascular.

Actors (n=20) fluent in the relevant language performed the role of the COPD patients. Fluency was confirmed by native speakers. Where language skills allowed, actors were used in multiple countries (seven actors only performed in one country, eleven actors performed in two countries, and two actresses performed in 3 countries), increasing the consistency with which the cases were presented to the physicians. Many of the actors were either native to, or had spent significant time living in the countries where the study was conducted. In addition, they spent time with local COPD patients to ensure they portrayed a COPD patient as socially and linguistically accurately as possible. The actors were trained in clinical aspects of COPD, details of their case and not to proactively raise their ‘patient issues’ with the physician, who needed to uncover them by direct enquiry.

Using actors to portray standardised cases provides confidence that differences seen in a study are due to the intervention, rather than differences in case presentations, especially as the study needed to run over several countries and several months. The use of trained actors to play standardised patients has been successfully used before in other studies assessing the behaviours of physicians [14,15], and by the Royal College of General Physicians in the assessment of primary care physicians in the UK. There is often a concern that simulated surgeries compromise the realism of the case, and the behaviour of the physician may be altered by knowledge of being observed (Hawthorne effect). However, physicians frequently report face-validity of patient actors [15], and the use of simulated consultations for the testing of an instrument’s properties is widely accepted and considered useful [16]. Additionally any impact of loss of realism could reasonably be expected to be similar for physicians in both arms.

### Assessment criteria

Independent assessors (n=24) with experience of assessing physician performance (e.g. through primary care qualification assessments, physician education programs), had experience of COPD management, but who were not part of the development of the CAT, assessed the videoed consultations. German and UK assessors also assessed Austrian and Irish physicians respectively (Table 1).

**Figure 3:**
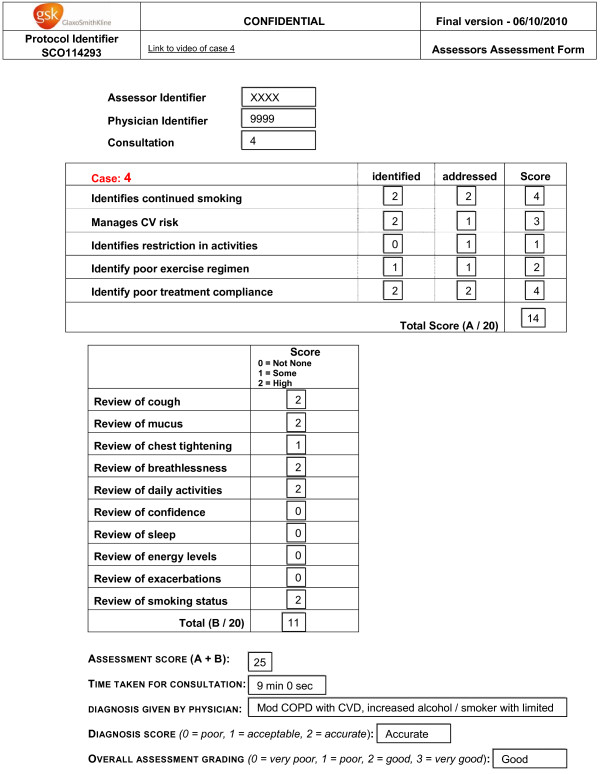
**Example assessment sheet.** Independent assessors reviewed the videoed consultations, and scored the physician on their ability to identify patient issues, review standard COPD symptoms, understanding of the case and overall performance.

All independent assessors were trained by HM through individual or small group telephone discussions. Assessors scored each physician on whether they identified and addressed the relevant ‘patient issues’ and reviewed ten standard COPD aspects such as cough and smoking history (0 = none, 1 = some, 2 = high). Scores were captured on an online score sheet, and the patient issues scores (sub-score A, out of 20) and COPD review scores (sub-score B, out of 20) were calculated. A global score of sub-score A plus sub-score B was calculated as a composite endpoint (Figure 3). A ‘some’ score for sub-score A and sub-score B was given when the physician had gained a superficial appreciation of the patient issue; while a ‘high’ score was awarded when the physician asked a number of insightful questions to fully understand the issue, the burden on the patient and how the situation might be improved. The independent assessors also rated the physician’s understanding of the case from their description to camera (“understanding score”: poor, acceptable, accurate) and their overall performance (very poor, poor, good, very good). Similar to the sub-score grading scheme, an ‘acceptable’ understanding score was given when the physician had understood the key elements of the case, while an ‘accurate’ score was awarded when the physician demonstrated a deep and full understanding of the case. Overall performance summarised the whole consultation including, for example how the physician behaved in the consultation, empathy etc.

Although the assessors from UK and Germany were trained to judge Irish and Austrian physicians in the same way as UK and German physicians, it is possible that their assessment differed due to cultural or health care system differences though it is unlikely that any impact from these differences would be different in the two arms.

These assessment criteria were prospectively created specifically for this study to test the utility of the CAT in the areas that we believed it might impact. They were not tested for assessor interpretation, inter- and intra- assessor consistency of marking or sensitivity (relative importance of each item on the quality of the consultation). The global score of sub-score A plus B was chosen as the primary endpoint as this is the most conservative endpoint, so we were not setting up the study for guaranteed success.

Alternative methods of assessing physician behaviour have been described previously [16-18]. The Consultation Quality Index-2 (CQI-2) measures the holistic quality of the consultation, including patient enablement, empathy and patient satisfaction [18], and so may not have been sufficiently sensitive to measure the impact upon a consultation that we expected the CAT to achieve. Similarly, the Roter’s Interaction Analysis System (RIAS) [17] focuses on the physicians psychosocial behaviour and quality of management of the patient:physician relationship, and as such may miss the expected impact of the CAT. The Medication-Related Consultation Framework (MRCF) tool assesses medication related consultations and focuses on the discussion content between a patient and pharmacist [16]. This tool includes sections on “data collection and problem identification” and “actions and solutions”, with the questions primarily assessing the patient:pharmacist discussion around medication use. The questionnaire is quite long (46 items), aimed at pharmacists, and - as medication compliance is only a small part of primary care consultations – would miss other discussion that the CAT may influence. We therefore felt that none of these established consultation assessment tools would have provided a sound basis of assessment of the impact of the CAT in the primary care setting.

Actor feedback is frequently used in studies of physician behaviour and is often found to be predictive of real patient feedback [15], with acceptably small variance between different actors [19]. Therefore the actors provided feedback following each consultation on whether they felt the physician addressed their issues by scoring 5 questions (such as “I felt the physician understood my issues”, “The physician helped me address my issues”) as “no”, “yes, but unsatisfactory” or “yes”. These questions were based on a similar questionnaire used to gather feedback on the healthcare service provided to UK COPD patients. The actors also provided feedback on the length of the consultation, and their overall satisfaction.

### Feasibility

We conducted a pilot study to confirm the feasibility and inform the sample size of the study. The methodology as described above was used with ten UK Physicians. Only minor technical issues were identified with the recruitment of physicians, consultation filming, and assessments, which were resolved in time for the main study.

The assessment of the physicians in the pilot study was conducted by some of the authors (SH, JR, GN and DL). The mean global score across the cases ranged between 12.8 and 19.0 in the *no CAT* arm, and 16.0 and 25.3 in the *CAT+* arm (Table 3); and the difference between the arms, ranged between 3.2 and 12.3. The percent of “good” or “very good” consultations rose by up to 40 percentage points in the *CAT+* arm.

**Table 3:** Pilot study results

**Case**	**NO-CAT**	**CAT+**
	**mean score (SD)**	**mean score (SD)**
**Case 1**	13.3 +/− 4.0	22.0 +/− 10.1
**Case 2**	13.0 +/− 5.6	25.3 +/− 6.4
**Case 3**	12.8 +/− 2.2	16.0 +/− 5.0
**Case 4**	16.0 +/− 9.7	19.3 +/− 11.9
**Case 5**	13.8 +/− 9.4	18.5 +/− 9.3
**Case 6**	19.0 +/− 9.5	24.6 +/− 8.2

Mean Global score from pilot study, by case. SD: Standard deviation.

The results from the pilot study indicate the assessment items and scoring system employed were sufficient to identify differences between good and poor consultations.

### Assessor scoring variability

A large number of independent assessors, from 3 different countries, were required to assess the volume of consultations which introduced significant complexity to the analysis of the study. Additionally, it was not possible to blind the assessors to the presence of the CAT from the consultation, which may have biased their scoring. Alternative methodologies, such as audio taped consultations, would not necessarily have resolved this. We conducted a benchmarking exercise to gauge whether these aspects would impact upon the final result of the study. The intention was to assess the variability in the assessors’ scores and to identify any outlying assessor(s). Each assessor therefore reviewed two high-scoring and two low-scoring consultations from the pilot study, with and without CAT.

**Figure 4:**
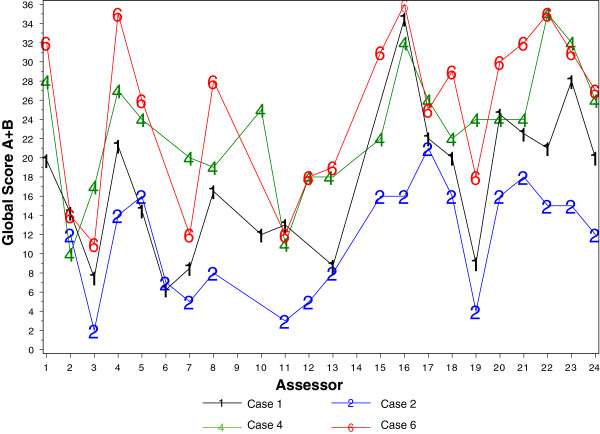
**Benchmarking assessment scores.** Each assessor reviewed 4 set cases, and the Global scores were compared to assess the variability of scoring between assessors, and to identify any outliers. Assessors reviewed a low scoring consultation with CAT (Case 1); a low scoring consultation without CAT (Case 2); a high scoring consultation without CAT (Case 4); a high scoring consultation with CAT (case 6).

The actual scores given by each assessor varied significantly, and a difference in mean total scores across the countries were identified; however the ranking and differences between the high and low scoring assessments were generally consistent (Figure 4). The inter-assessor reliability was analysed. The intraclass correlation coefficient (ICC) was 0.68 [95% CI 0.38; 0.97], p<0.001, and no atypical assessor was identified.

The benchmarking analysis also suggested that ‘case’ was as much a factor in the different scores as the assessor – i.e. variability in the actual score across the assessors was not obscuring the difference between the cases. To account for these effects, assessor and case were included as adjustment variables in the statistical analyses.

### Statistical aspects

The primary endpoint for the study was mean global score (combined sub-scores A and B, which had a scale of 0 (worst) to 40 (best)). For the one case which only had four patients issues, compared to the other cases with five, sub-score A results were scaled up (multiplied by 1.25) to make it comparable. Since the primary endpoint had not been previously trialled, the sensitivity or potential magnitude of difference between the arms was unknown. The investigators agreed that a difference of 10% (i.e. 4 points) between the arms would be convincing as a true difference. The pilot study data indicated that this difference was realistic, and that a conservative estimation of standard deviation was 12 points. Therefore, in order to achieve 90% power to detect a difference of at least 3 points in global score in the main study 752 consultations were needed, allowing for 10% missing data. No adjustments for confounders were included. Given the number of patient consultations created at each session and location, the number of planned patient consultations was 960.

Sub-scores A and B, were also designated as secondary outcomes; all scores were analysed using repeated-measures analysis of variance with a linear mixed model. Differences in items of global score, and of sub-scores A and B were tested with a chi-square test or a Fisher’s exact test. ‘Understanding score’ and ‘overall assessment’ grading were analysed with a generalized estimating equations model. To account for the assessor effect, as identified in the benchmarking exercise, both assessor and case were included as adjustment variables in the models. The order in which physicians saw the cases was also included to account for any training effect. A secondary analysis where physician characteristics, including country, were explored as potential confounders was conducted to confirm the generalisability of the study results across multiple countries. Similarly a sensitivity analysis including all physicians who participated in the study was conducted. The statistical analysis was conducted using SAS v9.1.

### Concluding remarks

This novel study was designed to assess the impact of the CAT on the behaviour of a physician in their consultation with a COPD patient. At the time the study was run there was no guidance available on managing COPD patients based on their CAT scores. As such we were not able to assess whether the CAT impacted the therapy and management choices made by the physician and patient. This situation has subsequently changed as CAT is now forms part of the Global Initiative for Chronic Obstructive Lung Disease (GOLD) 2011 COPD assessment framework, alongside spirometry and exacerbation history [20]. Additionally, this methodology would not be able to evaluate the impact of CAT on long-term management and outcomes. Further studies on the CAT specifically are warranted.

To ensure a fair comparison between the arms in this study, we needed to take steps to ensure that potential confounders such as underlying knowledge of the physicians, suitability of the assessment criteria, and variability of the assessors were as controlled as possible. Although there are still some limitations, the study design allows standardisation of the cases and analysis, and could be used to assess the utility of other PROMs by direct observation of clinical practice.

## Abbreviations

CAT: COPD Assessment Test; COPD: Chronic Obstructive Pulmonary Disease; CQI-2: Consultation Quality Index-2; ICC: Intraclass correlation coefficient; MRCF: Medication-Related Consultation Framework; PROM: Patient reported outcome measure; RIAS: Roter’s Interaction Analysis System; SGRQ: St George’s Respiratory Questionnaire; UK: United Kingdom.

## Competing interests

KGJ has acted as a consultant for and spoken on behalf of GSK, AZ, Chiesi, Boehringer Ingelheim, MSD, Muni Pharma/Napp, Allmirall, Novartis, Sandoz. SH has received speaker fees, travel grants and honoraria for advisory board from AZ, Boehringer Ingelheim, Chiesi, GSK, MSD, Napp, Novartis and Nycomed. PK has received honoraria for advisory board, travel grants and speaker fees from AZ, Boehringer Ingelheim, Chiesi, GSK, MSD, Novartis, Nycomed RE has received honoraria for advisory board, travel grants and speaker fees from AZ, Boehringer Ingelheim, Chiesi, GSK, MSD, Novartis, Nycomed RDN reports no conflict of interest JR has received speaker fees, travel grants and honoraria for advisory board from AZ, Boehringer Ingelheim, Chiesi, GSK, MSD, Novartis, Teva. PJ has received fees from pharmaceutical companies, including GlaxoSmithKline, for speaking at meetings and participating in advisory board meetings, and has received support for research from pharmaceutical companies, including GlaxoSmithKline. HM, GN and DL are employees of GlaxoSmithKline, who funded this study.

## Authors’ contributions

All authors heavily inputted into the design of the study, from designing the patient cases and assessment criteria to defining the endpoints and statistical analysis used in the study. HM wrote this paper and all other authors have critically reviewed and approved it. Additionally, KGJ, SH, PK, RE, JR, GN and DL assisted in the assessment of the consultations, and PJ assisted design the COPD patient cases. All authors read and approved the final manuscript.

## Pre-publication history

The pre-publication history for this paper can be accessed here:

http://www.biomedcentral.com/1471-2288/13/63/prepub
